# The Relation between Oral *Candida* Load and Bacterial Microbiome Profiles in Dutch Older Adults

**DOI:** 10.1371/journal.pone.0042770

**Published:** 2012-08-10

**Authors:** Eefje A. Kraneveld, Mark J. Buijs, Marc J. Bonder, Marjolein Visser, Bart J. F. Keijser, Wim Crielaard, Egija Zaura

**Affiliations:** 1 Department of Preventive Dentistry, Academic Centre for Dentistry Amsterdam, University of Amsterdam and VU University Amsterdam, Amsterdam, The Netherlands; 2 Department of Health Sciences, VU University Amsterdam, Amsterdam, The Netherlands; 3 Microbiology and Systems Biology, TNO Earth, Environmental and Life Sciences, Zeist, The Netherlands; Louisiana State University, United States of America

## Abstract

Currently there are no evidence-based ecological measures for prevention of overgrowth and subsequent infection by fungi in the oral cavity. The aim of this study was to increase our knowledge on fungal–bacterial ecological interactions. Salivary *Candida* abundance of 82 Dutch adults aged 58–80 years was established relative to the bacterial load by quantitative PCR analysis of the Internal Transcribed (ITS) region (*Candida*) and 16S rDNA gene (bacteria). The salivary microbiome was assessed using barcoded pyrosequencing of the bacterial hypervariable regions V5–V7 of 16S rDNA. Sequencing data was preprocessed by denoising and chimera removal, clustered in Operational Taxonomic Units (OTUs) and assigned to taxonomy. Both OTU-based (PCA, diversity statistics) and phylogeny-based analyses (UniFrac, PCoA) were performed. Saliva of Dutch older adults contained 0–4 × 10^8^ CFU/mL *Candida* with a median *Candida* load of 0.06%. With increased *Candida* load the diversity of the salivary microbiome decreased significantly (*p*<0.001). Increase in the *Candida* load correlated positively with class Bacilli, and negatively with class Fusobacteria, Flavobacteria, and Bacteroidia. Microbiomes with high *Candida* load were less diverse and had a distinct microbial composition towards dominance by saccharolytic and acidogenic bacteria - streptococci. The control of the acidification of the oral environment may be a potential preventive measure for *Candida* outgrowth that should be evaluated in longitudinal clinical intervention trials.

## Introduction

In the next decades healthcare services are faced with an aging population. In the European Union the proportion of 65 year and older is predicted to reach 53% of the total population by the year 2025 [1]. An aging population forms a health risk group due to several factors. First, there are internal factors such as senescence of tissues (*e.g.*, mucosal fragility) and senescence of functions (*e.g.*, lowered immune response). Secondly, there are extrinsic factors such as polypathologies (*e.g*., diabetes and malignancies), polymedications and malnutrition [2]. The internal and external factors, which contribute to changes during aging, may also disturb the balance in the oral microbial ecosystem. A disturbance in the oral homeostasis between bacteria and fungi (*e.g*., *Candida*) may cause oral infectious disease.

*Candida* species are a part of the commensal oral microbiota of healthy individuals at all ages with a reported prevalence between 15–75% [3], [4] up to 80% in elderly, especially denture wearers [5], [6]. The use of antibiotics and corticosteroids, chemotherapy, malnutrition, premature birth and old age are among the most common predisposing factors for opportunistic fungal infections *i.e.* mycoses [7]. The incidence of invasive fungal infections is on the rise. A study of the epidemiology of sepsis in the U.S. found that the annual number of cases of sepsis caused by fungal organisms increased by 207% between 1979 and 2000 [8]. Among the nosocomial bloodstream infections recorded in the U.S. between 1995 and 2002, 9.5% were fungal in origin with *Candida* spp. being the fourth leading cause of all cases [9]. Furthermore, the burden of invasive mycosis is shifting from the intensive care unit to the general hospital and even outpatient setting, where most types of mycosis represent endogenous infection in which the normally commensal host microbiota take advantage of the “opportunity” to cause infection [7].

Recent advances in research on bacterial–fungal inter-kingdom communication have shown that bacteria are capable of interfering with or supporting the surrounding fungal community and *vice versa* [10], [11], [12]. It is expected that novel therapeutics will be based on “jamming” quorum sensing among pathogens and supporting communication between beneficial microbes. For practical reasons these studies are focusing on bacterial-fungal interactions *in vitro* at oversimplified conditions and with only a few species involved. No efforts so far have been made to assess the ecology and commensal relation of *Candida* with the oral microbiota at the breadth of the oral microbiome. There is an acute need to enhance our knowledge on the ecology of fungal – bacterial interactions in search for potential targets for a guided ecological balance towards a healthy oral ecosystem.

The specific aim of this study was to assess the relationship between the load of oral *Candida* and the diversity and composition of the oral bacterial microbiome. For this we obtained oral bacterial pyrosequencing profiles of healthy Dutch older adults with various levels of oral *Candida* carriage and established that there is a relationship between the oral *Candida* load and the bacterial microbiome.

## Results

### Overall Sequencing Output

Of all reads (available at SRA of NCBI as SRA050301), 90% passed the quality control, 73% passed denoising and 59% reads (805413 reads; average length 419 nt) remained after removing the reads with chimeric sequences. The 601 unique sequences clustered into 425 OTUs of which 334 OTUs contained at least 5 reads. On average, a sample contained 87 (SD 39; min 21, max 222) OTUs. In order to avoid the effect of variable sample size on the diversity analyses, a randomly picked subset of 800 reads per sample was created and used for all subsequent analyses. After random picking of the subset of 800 reads per sample, 300 OTUs remained in the dataset with an average 43 (SD 13; min 11, max 73) OTUs per sample (Dataset S1). The subsampled reads were classified into 12 phyla (Table S1), with Firmicutes dominating the dataset (57% of reads), followed by Actinobacteria (20%), Bacteroidetes (14%) and Proteobacteria (7%). The predominant genera were *Streptococcus* (34% of reads), *Rothia* (12%), *Veillonella* (11%), *Prevotella* (10.5%), *Gemella* (6.9%), *Actinomyces* (6.7%), *Neisseria* (4.5%), *Porphyromonas* (2.6%), *Haemophilus* (1.8%), *Lactobacillus* (1.2%) and *Leptotrichia* (1% of reads) (Dataset S2).

### Individual Microbiomes by Candida Load

**Figure 1:**
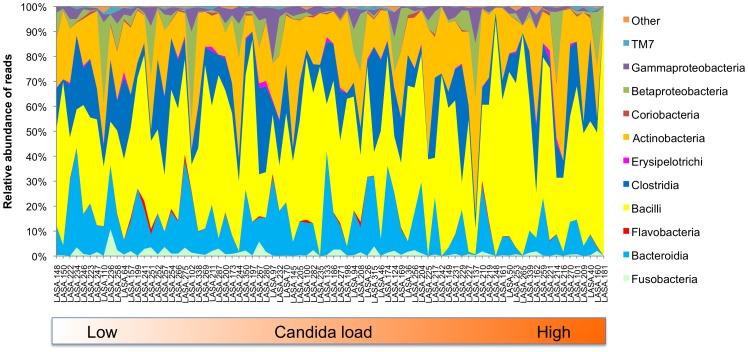
Relative abundance of bacterial taxa in saliva samples at the class level. The sample order corresponds to the increase in *Candida* load in the samples (Table S2). The *Candida* load was measured as proportion of ITS gene over 16S gene abundance by qPCR. Increase in *Candida* load correlated positively with reads classified as Bacilli (*p* = 0.013; Spearman’s rho 0.274), while class Bacteroidia (*p* = 0.001; Spearman’s rho −0.360), Flavobacteria (*p* = 0.003; Spearman’s rho −0.322), Fusobacteria (*p* = 0.012; Spearman’s rho −0.275) correlated negatively with the *Candida* load (Figure S1).

Saliva of Dutch older adults contained between 0 and 14.5% *Candida* as a proportion of *Candida* specific ITS gene over bacterial DNA (16S rRNA gene), with a median *Candida* load of 0.06% (Table S2). The exception was a single sample (LASA181) where *Candida* genes dominated over bacterial 16S gene at more than three orders of magnitude (ITS/16S = 1440%). The microbial profiles of these samples showed a high interindividual variation already at the bacterial class level (Figure 1). Increase in *Candida* load correlated positively with reads classified as Bacilli (*p* = 0.013; Spearman’s rho 0.274), while class Bacteroidia (*p* = 0.001; Spearman’s rho −0.360), Flavobacteria (*p* = 0.003; Spearman’s rho −0.322) and Fusobacteria (*p* = 0.012; Spearman’s rho −0.275) correlated negatively with the *Candida* load (Figure S1). Sample LASA181 differed in the microbial composition from the rest of the samples by dominance of genus *Lactobacillus* (81% of reads) and *Streptococcus* (16% of reads). This particular sample was the abovementioned outlier also regarding the *Candida* load.

**Figure 2:**
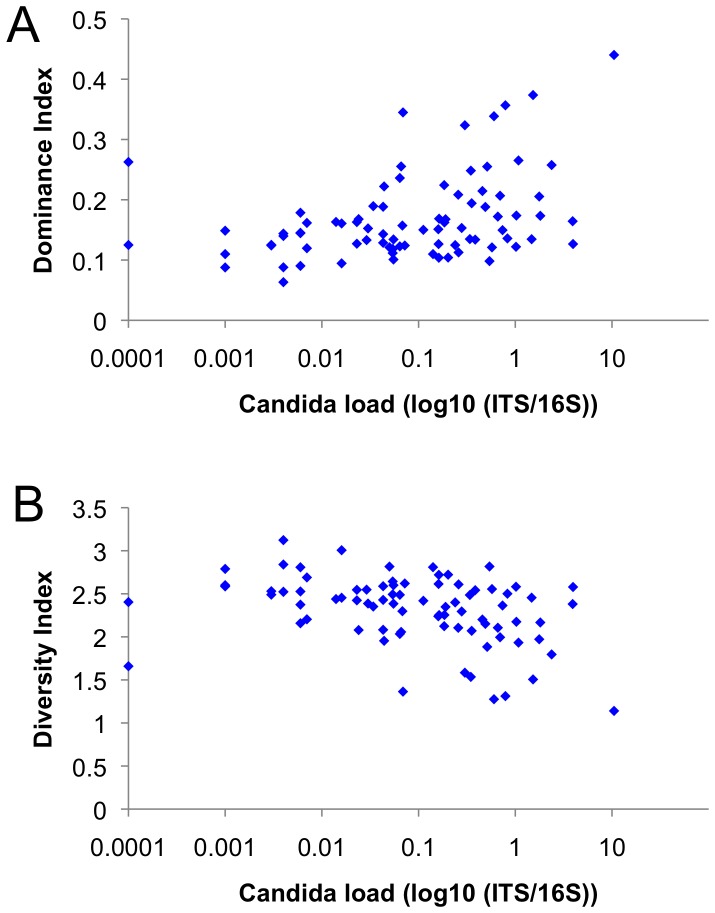
Diversity statistics of salivary microbiomes by *Candida* load. Diversity statistics of salivary microbiomes as: A) Dominance Index and B) Shannon Diversity Index by log10 *Candida* ITS/16S gene. *Candida* load correlated positively with the Dominance Index (*p*<0.001; Spearman’s rho 0.388) and negatively with the Shannon Diversity Index (*p*<0.001; Spearman’s rho −0.403).

Diversity statistics showed that the *Candida* load correlated positively with the Dominance Index (*p*<0.001; Spearman’s rho 0.388) and negatively with the Shannon Diversity Index (*p*<0.001; Spearman’s rho −0.403) (Figure 2) and the number of OTUs per sample (*p* = 0.003; Spearman’s rho −0.321). To assess the relation of the *Candida* load with the individual microbiome, the samples were divided into three categories - low (<0.01% *Candida*), medium (0.01–0.1%) and high *Candida* load (>0.1%) groups. The samples with a high *Candida* load were less diverse than samples with low *Candida* (*p*<0.005) (Figure S2C).

**Figure 3:**
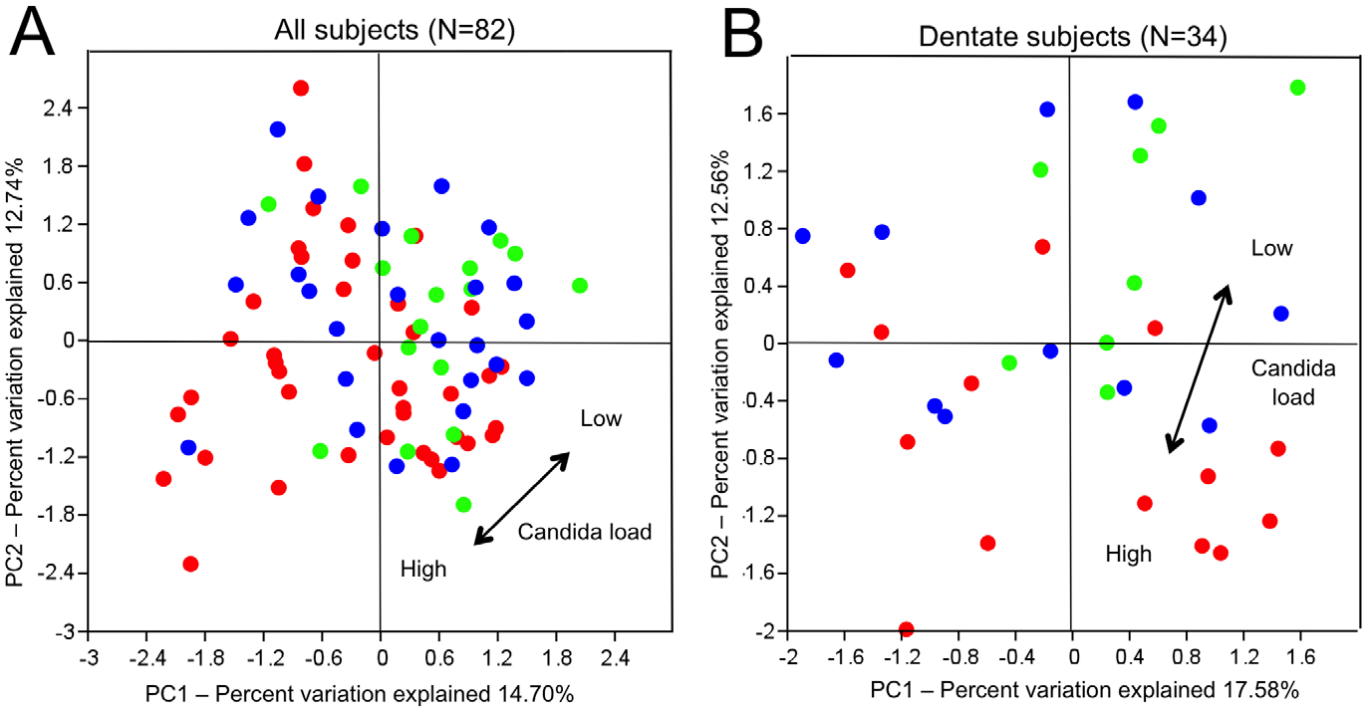
Salivary microbiome data by *Candida* load. Salivary microbiome data is plotted as OTU-based Principal Component Analysis (PCA) plot by *Candida* load in A) all subjects (N = 82) and in B) dentate subjects only (N = 34). Samples are colored by *Candida* load: green - low, blue - medium and red - high *Candida* load. Arrow indicates the main PC direction between low and high *Candida* load samples.

The multidimensionality of the data was first reduced by an OTU-based method – Principal Component Analysis (PCA). There was no clear separation into sample clusters by the *Candida* load. Rather than that, a continuous gradient from low to high *Candida* load samples was observed (Figure 3A, arrow). Next, we applied the phylogeny-based method UniFrac to the data. UniFrac accounts for the phylogenetic divergence between the OTUs. No separation of samples was obtained in principal coordinate analysis (PCoA) on unweighted (qualitative) UniFrac distances (Figure S3A), while weighted (quantitative) UniFrac distances (Figure S3B) showed a gradient from low to high *Candida* load samples similar to the PCA plot.

Based on the loadings of the PCA, the OTUs that contributed most towards the low *Candida* load sample direction in the PC1 were classified as *Prevotella* (4 OTUs), *Actinomyces*, *Veillonella*, *Megasphaera*, *Leptotrichia* (2 OTUs) and in the PC2– *Porphyromonas*, *Neisseria*, *Haemophilus* and *Prevotella*. The OTUs that contributed towards high *Candida* load sample direction were classified as *Streptococcus* (2 OTUs), *Lactobacillus* (2 OTUs), *Rothia* and *Gemella*. Prevalence of OTUs classified as *Lactobacillus* (2 OTUs) and *Scardovia* were associated with the high *Candida* load (*p*<0.005, G-test, NS after Bonferroni correction). Abundance of OTUs classified as candidate division TM7, *Leptotrichia* (2 OTUs), *Prevotella* (3 OTUs), *Peptostreptococcus*, *Capnocytophaga* and *Johnsonella* were associated with the low *Candida* load (*p*<0.005, ANOVA, NS after Bonferroni correction).

### Individual Microbiomes by the Presence of Teeth versus Full Dentures

From the information obtained by the questionnaire we could split the subjects into a dentate (N = 34) and an edentate (N = 20) group (Table S2). The edentate subjects reported regular use of full upper and lower dentures. The dentate subjects did not report wearing any prosthesis. The remaining subjects were either partially dentate and wore partial prosthesis (N = 20) or did not fill the questionnaire completely and were classified as “Not reported” (NR) (N = 8).

Diversity statistics showed that samples from dentate subjects had significantly more OTUs per sample (mean 47, SD 11) than samples from the edentate group (mean 34, SD 14) (*p* = 0.001, Mann-Whitney test) and had higher sample diversity (Shannon Diversity Index 2.4, SD 0.3) than subjects with full dentures (2.1, SD 0.5) (*p* = 0.021) (Figure S2 D–F).

Although dentate subjects showed a trend for a lower *Candida* load (median 0.04%, mean 0.32%, SD 0.6) than edentate subjects (median 0.16%, mean 72.6%, SD 322), there was no statistically significant difference in the *Candida* load between these groups (*p* = 0.311, Mann-Whitney test).

The three data reduction approaches - the OTU-based PCA and the PCoA of the unweighted and the weighted UniFrac distances (Figure S4) - all stratified samples into the dentate and the edentate groups. The loadings of the PCA showed that *Prevotella* (4 OTUs), *Actinomyces*, *Leptotrichia* and *Neisseria* contributed towards the dentate group, while *Rothia*, *Lactobacillus* (3 OTUs) and *Streptococcus* (3 OTUs) contributed to the edentate group.

Subjects with a dentition had a higher prevalence of OTUs classified as genus *Parvimonas, Peptostreptococcus*, unclassified *Micrococcales*, *Prevotella*, *Solobacterium*, *Neisseria* and *Abiotropia* (*p*<0.05; G-test; NS after Bonferroni correction) and a higher abundance of OTUs belonging to genus *Prevotella*, *Leptotrichia, Peptostreptococcus*, *Parvimonas*, *Solobacterium, Dialister* and *Porphyromonas* (p<0.05; ANOVA; not significant (NS) after Bonferroni correction). Edentate subjects had a higher prevalence of *Lactobacillus* (*p*<0.05; G-test; NS after Bonferroni correction) and a higher abundance of an OTU classified as *Rothia* (*p*<0.05; ANOVA; NS after Bonferroni correction).

### Individual Microbiomes of the Dentate Group by Candida Load

**Figure 4:**
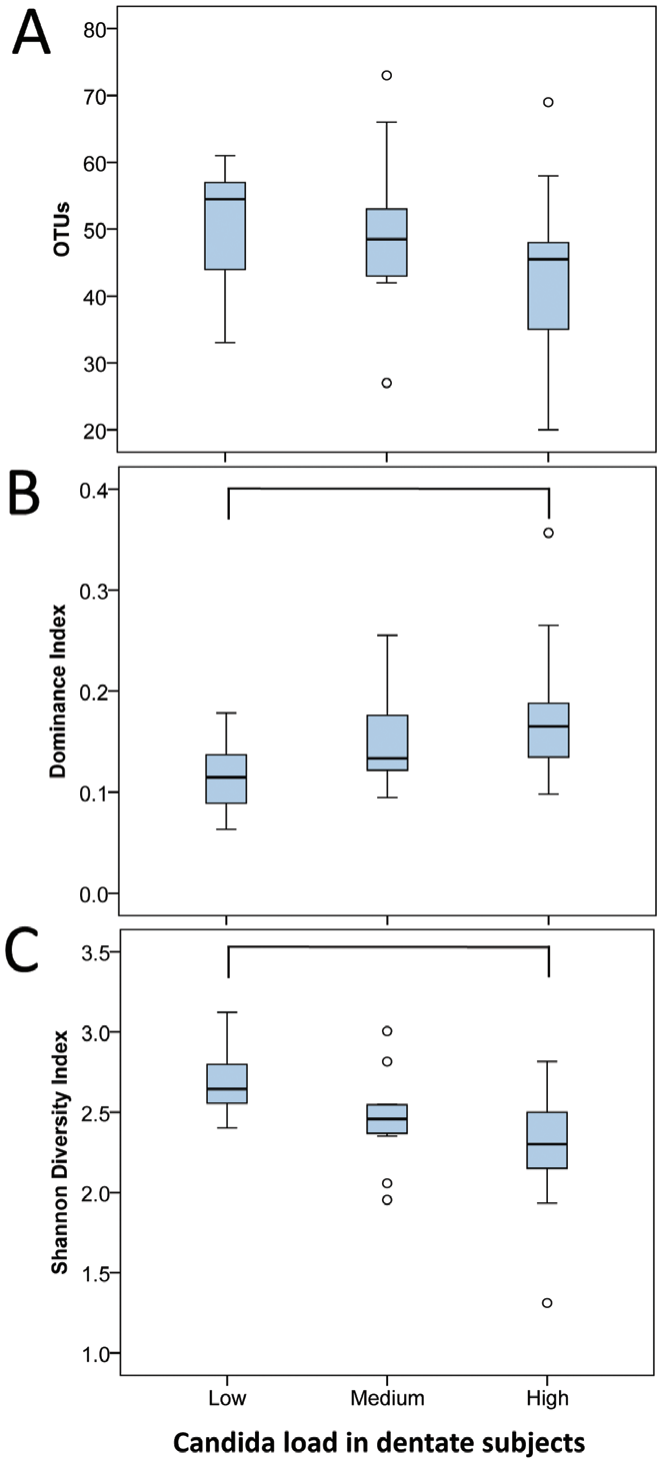
Diversity statistics by *Candida* load in dentate subjects. Diversity statistics by *Candida* load (low (N = 8), medium (N = 12), high (N = 14)) in dentate subjects as boxplots of: A) OTUs per sample, B) Dominance Index and C) Shannon Diversity Index. Each box shows the median, quartiles, and outliers (circles). Connector connects statistically significantly different groups (*p*<0.05; Mann-Whitney test).

To exclude the covariance of dentures on the relation of the *Candida* load with the microbiome profiles, we used the dentate group (N = 34) alone. The diversity statistics (Figure 4) showed that among the dentate subjects, as with the entire study group, the samples with a high *Candida* load (N = 14) were less diverse (Shannon Diversity Index 2.3, SD 0.4) than samples with low *Candida* (N = 8) (mean 2.7, SD 0.2) (*p* = 0.005, Mann-Whitney test). Additionally, the Dominance Index was significantly higher in the high *Candida* load samples (*p* = 0.012).

The PC 1 (18% of variance) and PC 2 (13% of variance) of the OTU-based PCA positioned most of the low *Candida* load samples and some medium load samples in the upper right quadrant of the PCA distant from the high *Candida* load samples (Figure 3B). The PCoA of the unweighted UniFrac distances showed that samples with a low *Candida* load were less dispersed than medium and high *Candida* load samples (Figure S3C). Weighted UniFrac (Figure S3D) separated low and high *Candida* samples in a similar way to the PCA (Figure 3B). Loadings of the PCA showed that genus *Streptococcus* (4 OTUs) contributed to samples from the high *Candida* load, while *Prevotella* (3 OTUs), *Leptotrichia* (2 OTUs), *Neisseria* and *Porphyromonas* determined the position of the low *Candida* load samples. The low *Candida* load was associated with higher abundance of OTUs classified as *Prevotella, Porphyromonas* and *Haemophilus* (*p*<0.05; ANOVA; NS after Bonferroni correction).

## Discussion

In this study we assessed the relation between the *Candida* load and the bacterial profiles of saliva of Dutch older adults. With increased *Candida* load the diversity of the salivary microbiome decreased and the composition changed towards dominance by Bacilli (streptococci and lactobacilli) and disappearance of genera within class Fusobacteria and Bacteroidia. The microbiome of dentate subjects differed from that of the fully edentate, but this difference was not significantly associated with the *Candida* load.

Saliva samples of our study group showed a large variation in *Candida* load: 0–4×10^8^ CFU/ml with a median *Candida* load of 0.06%. Studies on comparable population report about 0.02% *Candida* in saliva, detected by culturing [5], [13], [14]. All but two (97%) of our samples were *Candida*-ITS gene positive. Studies on a similar age group, though using a culturing approach, have reported around 80% *Candida* prevalence [5], [6]. This difference could be due to the lower detection limit and higher selectivity of the culturing technique compared with the qPCR on *Candida*-specific internal transcriber spacer (ITS) region, used here.

Due to the study logistics it was not possible to collect samples from defined intraoral habitats such as plaque, tongue or denture surfaces. Although different intraoral niches select for a habitat-specific microbiome and salivary microbial profiles show higher similarity to mucosal sites than to dental surfaces [15], saliva is frequently preferred as a representative oral sample for large clinical studies [16].

Dentures, especially in edentate individuals are known to be one of the main predisposing factors for outgrowth of *Candida* frequently leading to denture stomatitis [17]. In our study population we found only a trend for higher *Candida* load in edentulous full prosthesis wearers. The lack of statistical significance could be explained by the small sample size (20 edentulous individuals) and thus lack of statistical power, as well as by good oral and general health of the study participants. It has been shown that denture stomatitis is a multifactorial disease where poorly fitting dentures, continuously worn dentures, poor oral hygiene and reduced salivary flow all increase the ability of *Candida* to colonize the dentures and contribute to the etiology of the disease [14], [17].

Saliva profiles of Dutch older adults were composed of commensal bacteria commonly found in human salivary microbiomes [15], [16], [18]. Pathogenic species, such as enterobacteria, staphylococci and pseudomonas were found only in few subjects. Our study group was not representative of a Dutch elderly population, but consisted of non-institutionalized, self-supportive older adults with a good general health, which might explain the low prevalence of the nosocomial *i.e.* hospital-acquired pathogens. However, in two cases relatively high proportions of pathogenic species were found: staphylococci in a single sample (LASA161) contributed to 9% of the reads, and two *Pseudomonadacea* OTUs contributed to 11% of the reads in an another sample (LASA137). Interestingly, both of these cases belonged to high *Candida* load group. *Candida* has been previously co-associated with *Staphylococcus aureus* and *Pseudomonas* in human infections [11]. It has been shown that *C. albicans* physically interacts with *S. aureus* and differentially regulates specific virulence factors of *S. aureus in vitro* [19].

We observed a negative correlation between the *Candida* load and the diversity of the salivary microbiome. We also showed that absence of teeth and presence of full dentures as such already were related to decreased microbiome diversity, irrespective of the *Candida* load. Therefore the effects of *Candida* load were assessed also on the dentate subgroup. Decreased diversity was seen both in the entire study sample and in the subgroup of dentate subjects. Decreased bacterial diversity is associated with a disbalanced community, *e.g*., the salivary microbiome from caries-active individuals was shown to be less diverse than microbiome from caries-free individuals [20]. Perturbations such as prolonged low pH episodes reduce the diversity of microbial communities due to acidification of the environment [21]. The importance of equal sample size on accuracy of diversity analyses was recently emphasized [22]. To avoid the bias of under- or oversampling, we used randomly subsampled dataset to the lowest number of reads per sample for all analyses.

The proportion of certain bacterial classes such as Fusobacteria, Bacteroidia, and Flavobacteria correlated negatively with the *Candida* load. These taxa are generally less acid-tolerant than Bacilli, which was the only class that increased with the increasing *Candida* load. This suggests that the oral environment of a high *Candida* load samples was acidified, *i.e.* exposed to prolonged low pH episodes. Oral *Candida* isolates have been shown to be highly acid tolerant and acidogenic [23] and able to lower the pH of a culture during growth from pH 7.5 to pH 3.2 [24].

Within the class Bacilli, streptococci increased most with increased *Candida* load. The interactions between fungi and streptococci appear to be synergistic [25], [26]. The growth of *Candida albicans* is enhanced if co-cultured with oral streptococci in the presence of sucrose [27]. *Streptococcus sanguinis*, *Streptococcus gordonii*, *Streptococcus oralis* and *Streptococcus anginosus* are known to coaggregate with *C. albicans*, especially if the yeast cells are subjected to glucose starvation [26]. These streptococcal species are able to adsorb protein components from human saliva (basic, proline-rich proteins) that in turn promote *C. albicans* adhesion [28]. The adherence of *S. gordonii* is mediated through streptococcal cell surface polysaccharide receptors and polypeptide adhesins [29]. *Candida*, on the other hand, in addition to reducing the oxygen tension to levels preferred by streptococci, may provide growth stimulatory factors for the bacteria as a result of nutrient metabolism [11]. Next to streptococci, also lactobacilli were associated with high *Candida* load. While most lactobacilli are recognized as probiotics regarding *Candida* control [30], *Lactobacillus casei* was shown to stimulate germ tube growth – an important *C. albicans* virulence factor [30], [31]. Both, streptococci and lactobacilli are in general aciduric and acid tolerant microorganisms, often associated with dental caries pathogenesis [32].

A low *Candida* load was associated with among others, genus *Prevotella, Porphyromonas* and *Peptostreptococcus.* There are no comparable clinical reports regarding any relationships of these bacteria with fungi. In *in vitro* studies however, two putative periodontal pathogens – *Prevotella nigrescens* and *Porphyromonas gingivalis* – have been shown to exert a suppressive effect on *C. albicans* viability [33], while *P. gingivalis* and *Prevotella intermedia* inhibited *C. albicans* germ tube formation *in vitro* [31]. With respect to the complexity of the oral ecosystem, it is not likely that each of the taxa is interacting with *Candida* or is affected by it directly. Our finding of *Peptostreptococcus* together with *Prevotella* in low *Candida* load samples might be due to a symbiotic relationship between these two taxa. For instance, it has been shown that *Peptostreptococcus anaerobius* is nutritionally dependant from *Prevotella bovia* (two species involved in pathogenesis of bacterial vaginosis), where *P. bovia* provides amino acids required for the growth of *P. anaerobius* [34]. Most probably multiple intermediate interactions among different metabolically dependent bacterial taxa as well as environmental conditions are affected by *Candida* or are affecting the presence of *Candida*. The exact mechanisms should be investigated further, both in longitudinal clinical trials and in fundamental experimental research *in vitro*.

The current findings strongly suggest that acidification of the environment coincides with the high load of *Candida* and is the major ecological factor that perturbs the oral commensal microbiome leading to the shift towards an increase of aciduric microbiota and reduction of natural diversity of the bacterial microbiome. The actual sequential order of the events (*i.e*., what comes first – *Candida* outgrowth followed by acidification or primary acidification of the oral ecosystem due to other factors such as dry mouth or high carbohydrate intake which eventually may lead to increased *Candida* load) can only be elucidated in well-planned longitudinal studies.

Our results bring us to two potential preventive strategies. First, the pH-lowering potential as well as duration of the low pH episode after carbohydrate consumption could be diminished by enrichment of base-producing bacteria. This could be achieved by modifying a diet from a carbohydrate- to an arginine-rich protein diet that could enhance the proportion of alkali-producing microbiota. Supplementation with prebiotics such as arginine or urea [35], [36] is also known to result in base production and counteracting acidification. So far clinical tests with these supplements have been focused on the inhibition of dental caries in a young and healthy population [37] and have not involved an older adult population at risk for *Candida* infection. Second, the finding of inverse relationship of certain taxa with the *Candida* load suggests a potential in search for and the use of natural, with oral health-associated probiotic strains, in fungi-bacteria warfare. These efforts will eventually bring us closer to developing strategies for prevention of microbial shifts and *Candida* overgrowth and to unraveling of and interfering with the interkingdom signaling processes.

### Conclusions

There is a relation between oral salivary *Candida* load and the bacterial microbiome profile in older adults. Microbiomes with a high *Candida* load are less diverse and have a distinct microbial composition predominated by streptococci. The actual role of *Candida* as opportunistic pathogen in shaping the microbiome should be determined in further research including fungal-microbial interactions in complex microcosm models *in vitro* and following natural interactions in healthy and diseased populations during longitudinal clinical trials.

## Materials and Methods

### Subjects and Saliva Collection

Study subjects (N = 82, mean age 66 years (SD 6.6); min 58, max 80 years; 50% females) were participants of the Longitudinal Aging Study Amsterdam (LASA) (http://www.lasa-vu.nl), a prospective study on predictors and consequences of changes in autonomy and well-being in the aging population in the Netherlands. The study was approved by the ethical review board of the VU University Medical Center (Amsterdam, the Netherlands), and all participants gave written informed consent. Subjects were chosen from three culturally distinct geographical areas in the West, Northeast, and South of the Netherlands. Details on the sampling and data collection procedures have been published elsewhere [38], [39]. From the 2165 participants who participated in the measurement cycle in 2005–2006, 1421 participants were selected who were <80 years of age, lived independently, had a good cognitive status (MMSE score>23 [40]) and were alive on January 15, 2007 for a sub-study on lifestyle including oral health. All selected persons received an extensive lifestyle questionnaire in February 2007 by mail. A total of 1058 participants (response 74.5%) returned a completed questionnaire (N = 326 no response, N = 18 refused, N = 8 not able due to physical or newly developed cognitive problems, N = 11 deceased). Information on oral health and dental status, gender and age was collected. Saliva was collected by the participants in the morning before they had taken breakfast, medicine, smoked tobacco, brushed their teeth or used any mouth rinse. Exclusion criterion was the use of antibiotics within the 3 months before saliva collection. Subjects were asked to remove their dentures during collection of saliva. Unstimulated saliva was collected by drooling into a DNA-free, sterile vial for 5 minutes [17]. The tubes were sent to the laboratory, saliva was alliquoted and stored at −80°C.

### DNA Extraction

Salivary DNA was extracted as described previously [16]. In brief, 0.1 ml of samples was lysed in phenol in a bead beating procedure, using 0.25–0.5 mm diameter (for breaking up the yeast cells) and 0.1-mm diameter (for breaking up the bacterial cells) glass beads (Lab Services BV, the Netherlands). DNA was extracted with the AGOWA mag Mini DNA Isolation Kit (AGOWA, Berlin, Germany) and samples were stored at −20°C until further analysis.

### qPCR

Real-time qPCR was used to determine the concentration (CFU/ml) of *Candida* and bacteria in the saliva samples. Primers specific for the *Candida ITS* rDNA gene (Forward: CCTGTTTGAGCGTCRTTT; Reverse:

TTCTCCGCTTATTGATAT) modified from [41] were used to determine the *Candida* concentration. Primers and probe specific for the prokaryotic *16S* rDNA gene (Forward: TCCTACGGGAGGCAGCAGT; Reverse:

GGACTACCAGGGTATCTAATCCTGTT, Probe:

6FAM-CGTATTACCGCGGCTGCTGGCAC-BBQ) [42] were used to determine the bacterial concentration. Real-time qPCR was performed using a LC480-II light cycler (Roche Diagnostics, Switzerland). As internal control for PCR inhibition a qPCR on PhHV (Phocid herpesvirus type 1 gB gene) was performed in all reactions and DNA and primers where added according to Watzinger et al. (2004) [43]. Reactions were performed with 3 µl of DNA. The total reaction volume was 20 µl. Reactions contained a 2× PCR Probe Master Mix (Roche) or, in the case of *ITS* primers, a 2× SYBR Green PCR Master Mix (Roche). For *16S*, 7.5 pmol primers and 3.8 pmol probe, for PhHV 1.8 pmol primers and 0.4 pmol probe and for *ITS* 24 pmol of each primer was used. The conditions for the qPCR reaction were: an activation step of 10 min at 95°C followed by 50 cycles consisting of a denaturation step at 95°C for 30 sec, an annealing step at 60°C for 30 sec and an extension step at 72°C for 30 sec. Standard curves obtained from the overnight cultures of *Candida dubliniensis* and *Escherichia coli* were used to extrapolate DNA concentrations (CFU/ml) of the *Candida* (*ITS*) and bacterial (16S) concentration (CFU/ml) in the saliva samples, respectively. The CFU numbers were validated in saliva samples spiked with six different *Candida* overnight cultures (*Candida tropicalis, Candida albicans, Candida glabrata, Candida parapsilosis, Candida krusei* and *Candida kefyr*) and performing both, culturing of *Candida* and performing *Candida* specific qPCR.

### Amplicon Preparation and Pyrosequencing

Amplicon libraries of the small subunit ribosomal RNA gene V5–V7 hypervariable region were generated for each of the individual samples. PCR was performed using the forward primer 785F (GGATTAGATACCCBRGTAGTC) and the reverse primer 1175R (ACGTCRTCCCCDCCTTCCTC). The primers included the 454 Life Sciences (Branford, CT, USA) Adapter A (for forward primers) and B (for reverse primers) fused to the 5′ end of the 16S rDNA bacterial primer sequence and a unique 10 nt sample identification key.

The amplification mix contained 2 units of Pfu Ultra II Fusion HS DNA polymerase (Stratagene, CA, USA), 1 unit Buffer Pfu Ultra II [10x], including 2.0 mM MgCl_2_ (Stratagene), 240 µM dNTP (Fermentas GMBH, Germany) 0.5 µM of each primer. To each reaction 100 pg of DNA template was added. After denaturation (95°C; 2 min), 9 cycles were performed consisting of denaturation (95°C; 30 sec), annealing (53°C; 30 sec), and extension (72°C; 80 sec), followed by 23 cycles of denaturation (95°C; 30 sec), annealing (62°C; 30 sec), and extension (72°C; 80 sec). The amplicons were purified by means of the IllustraTM GFXTM PCR DNA and Gel Band Purification Kit (GE Healthcare, Eindhoven, the Netherlands). The quality and the size of the amplicons were analyzed on the Agilent 2100 Bioanalyser with the DNA 1000 Chip kit (Agilent Technologies, Santa Clara, CA, USA). The amplicon libraries were pooled in equimolar amounts and sequenced unidirectionally in the reverse direction (B-adaptor) by means of the Genome Sequencer FLX Titanium system (Roche, Basel, Switzerland).

### Sequencing Data Analysis

The sequencing data were processed using QIIME (Quantitative Insights Into Microbial Ecology) [44] version 1.4.0. For further downstream analyses, barcodes and primer sequences were trimmed and low quality reads (reads containing ambiguous base calls, >1 error in the primer, >1 error in the barcode, >5 nt homopolymer sequence, the average quality score below 25, or a length <150 bp or >1000 bp) were removed from the analyses. Sliding window test (50 nt) of quality scores was enabled and sequences of low quality were truncated at the beginning of the poor quality window. The reads were denoised using Denoiser version 1.3.0 [45]. The denoised reads were checked for chimeric sequences using UCHIME version 4.2.40 [46]. The results of the *de novo* and the reference-based approach were combined and reads marked as chimeric were removed.

The cleaned reads were clustered into Operational Taxonomic Units (OTUs) at a minimal sequence similarity of 97% using UCLUST Reference Optimal [47]. The representative sequence of each cluster was assigned a taxonomy using the Ribosomal Database Project (RDP) classifier [48]. The minimum confidence was set at 0.8. For taxonomy assignment the SILVA rRNA database [49] was trimmed to span the targeted hypervariable regions V5– V7 as described by Brandt et al. [50]. The taxonomy assigned OTUs were aligned using PyNAST [51]. For the alignment, the minimum aligned sequence length was set at 150 nt and the minimum percent identity at 75%. FastTree [52] was used to build a phylogenetic tree of the representative sequences.

To allow comparisons among different samples, a randomly subsampled dataset of 800 reads per sample (the minimum nr of reads per sample was 881) was created. Data reduction by principal component analysis (PCA) on log2 transformed OTU data and the diversity statistics (Shannon Diversity Index, Dominance Index) was performed using PAST software [53]. Shannon Diversity index takes into account the number of taxa (OTUs) and the relative contribution of each OTU to the whole dataset. Dominance Index measures the ‘dominance’ of the community and ranges from 0 (all taxa equally present) to 1 (one taxon dominates the community completely).

For phylogenetic measures of community ß diversity, unweighted UniFrac (a qualitative measure) and weighted UniFrac (a quantitative measure) [54] were used in QIIME. Principal coordinates analysis (PCoA), in which a distance matrix is used to plot the *n* samples in (*n* –1)-dimensional space, was used to compare groups of samples based on unweighted and weighted UniFrac distance metrics. PCoA is analogous to a PCA. The distinction is that PCA begins with a table of the number of times each OTU was observed in each environment, whereas PCoA begins with a table of distances between each pair of environments [54].

Spearman’s correlation was performed to assess the correlation between the proportion of *Candida* (% ITS/16S) in saliva and the abundance of individual bacterial classes, the Mann-Whitney test was used to compare diversity statistics between sample groups (high vs. low *Candida* load, dentate vs. edentate subjects) using SPSS version 17.0. A G-test of Independence and ANOVA (implemented in QIIME 1.4.0.) were used to identify OTUs that are differentially represented among the groups of samples. The G-test of independence determines whether presence/absence of an OTU is associated with a category. ANOVA determines whether the relative abundance of an OTU is different between categories. The categorical variables used were: high versus low *Candida* load; dentate versus edentate subjects; and within the dentate group – high and low *Candida* load. The medium *Candida* load was arbitrarily set at an ITS/16S gene proportion between 0.01% and 0.1%. All values below 0.01% were considered low, values above 0.1% – high *Candida* load. The probability after multiple comparisons was corrected using Bonferroni correction. In this correction the *p* value is multiplied by the number of comparisons (the number of OTUs remaining after applying the filter). The filter that was applied required that a minimum of 10 samples contained the OTU for the OTU to be included in the analysis.

## Supporting Information

Figure S1 **Relative abundance of four bacterial classes that showed correlation with** ***Candida*** **load plotted against the** ***Candida*** **load.** The abundance (nr of reads/sample) of (A) Bacilli, (B) Bacteroidia, (C) Fusobacteria and (D) Flavobacteria by *Candida* load. *Candida* load was measured as the proportion of ITS gene over 16S gene abundance by qPCR. The values were normalized by log10 transformation.
(TIF)

Figure S2 **Diversity statistics of salivary microbiomes by** ***Candida*** **load.** Diversity statistics of salivary microbiomes by *Candida* load (A–C) in saliva and by Dentition of the subjects (D–F) as boxplots of (A, D) OTUs per sample, (B, E) Dominance Index and (C, F) Shannon Diversity Index. Each box shows the median, quartiles, and outliers (circles). Connector connects statistically significantly different groups (*p*<0.05; Mann-Whitney test).
(TIF)

Figure S3 **Salivary microbiome UniFrac distance data by** ***Candida*** **load.** Salivary microbiome UniFrac distance data plotted by *Candida* load in all individuals (A, B) and in dentate individuals only (C, D) as unweighted (A, C) and weighted (B, D) UniFrac distance PCoA plots. Samples are colored by *Candida* load: green - low, blue - medium and red - high *Candida* load.
(TIF)

Figure S4 **Salivary microbiome data plotted by dentition.** Salivary microbiome data plotted by dentition of the subjects: A) PCA of OTU-based distances, B) PCoA of unweighted (qualitative) UniFrac distances and C) PCoA of weighted (quantitative) UniFrac distances. Samples are color-coded by the dentition status of the subjects: dentate (N = 34) – blue, edentate (N = 20) – red.
(TIF)

Table S1 Relative abundance of reads and number of OTUs per phylum.
(DOCX)

Table S2 Relative and absolute abundance of *Candida* (ITS gene) in saliva samples.
(DOCX)

Dataset S1 **OTU-table of salivary microbiome data.**
(XLSX)

Dataset S2 **Distribution of higher taxa (genus or higher taxon) in saliva samples.**
(XLSX)
