# Supplementary figures and images for: The Relation between Oral Candida Load and Bacterial Microbiome Profiles in Dutch Older Adults
Source: PLoS One. 2012 Aug 10;7(8):e42770. doi: 10.1371/journal.pone.0042770 (PMC3416775; doi:10.1371/journal.pone.0042770)

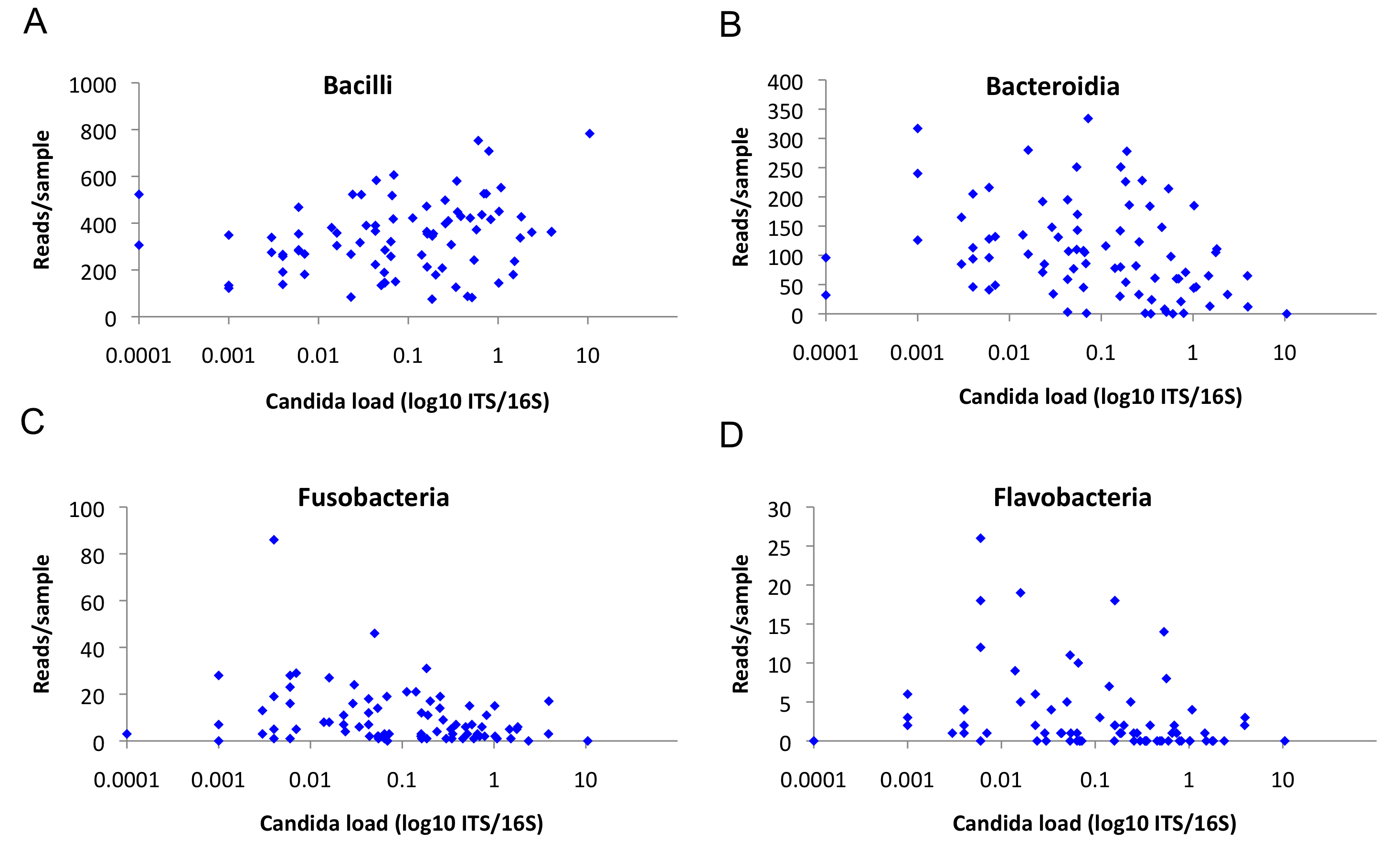

Supplement: Figure S1 — Relative abundance of four bacterial classes that showed correlation with Candida load plotted against the Candida load. The abundance (nr of reads/sample) of (A) Bacilli, (B) Bacteroidia, (C) Fusobacteria and (D) Flavobacteria by Candida load. Candida load was measured as the proportion of ITS gene over 16S gene abundance by qPCR. The values were normalized by log10 transformation. (TIF) [file pone.0042770.s001.tif]

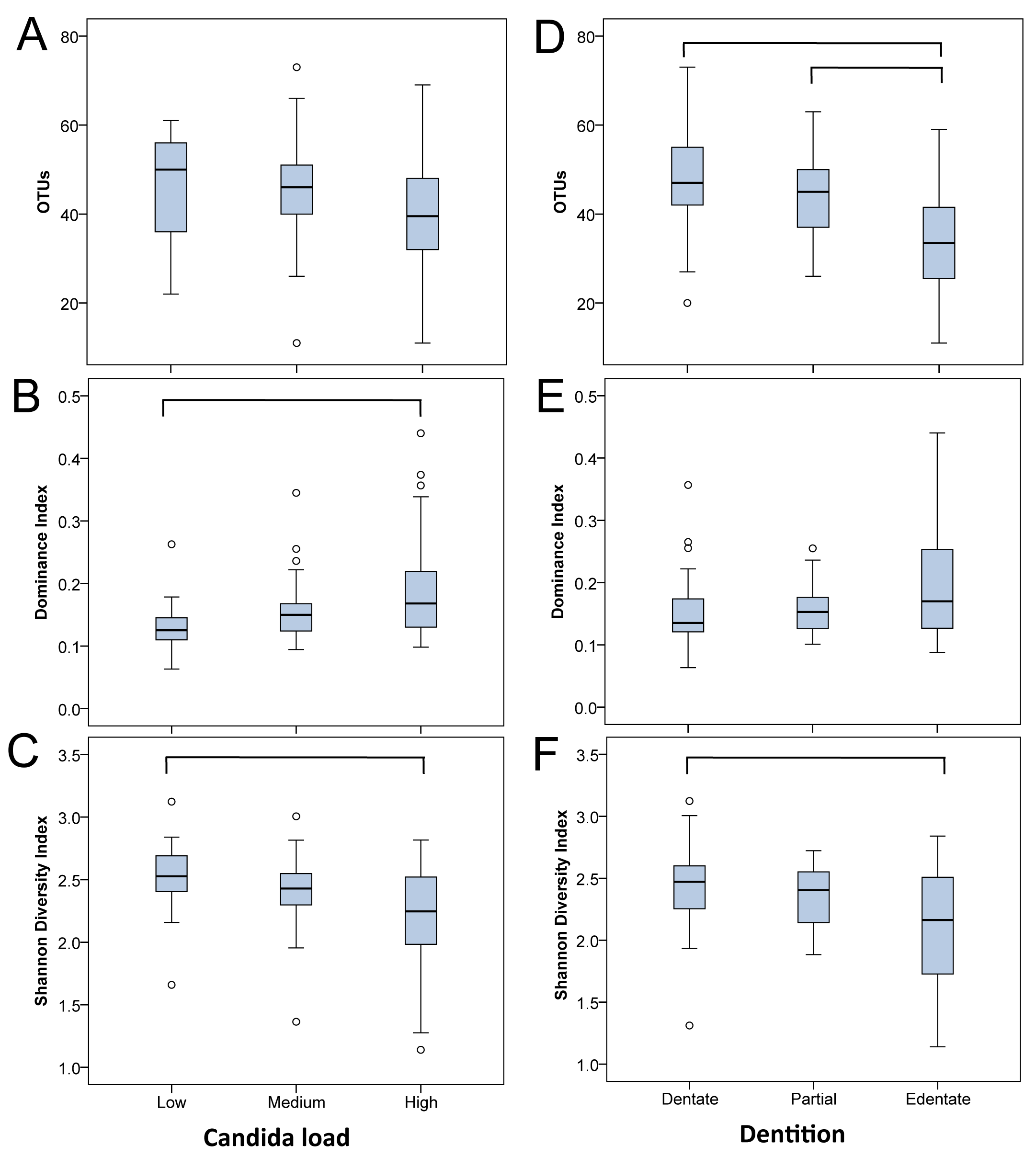

Supplement: Figure S2 — Diversity statistics of salivary microbiomes by Candida load. Diversity statistics of salivary microbiomes by Candida load (A–C) in saliva and by Dentition of the subjects (D–F) as boxplots of (A, D) OTUs per sample, (B, E) Dominance Index and (C, F) Shannon Diversity Index. Each box shows the median, quartiles, and outliers (circles). Connector connects statistically significantly different groups (p<0.05; Mann-Whitney test). (TIF) [file pone.0042770.s002.tif]

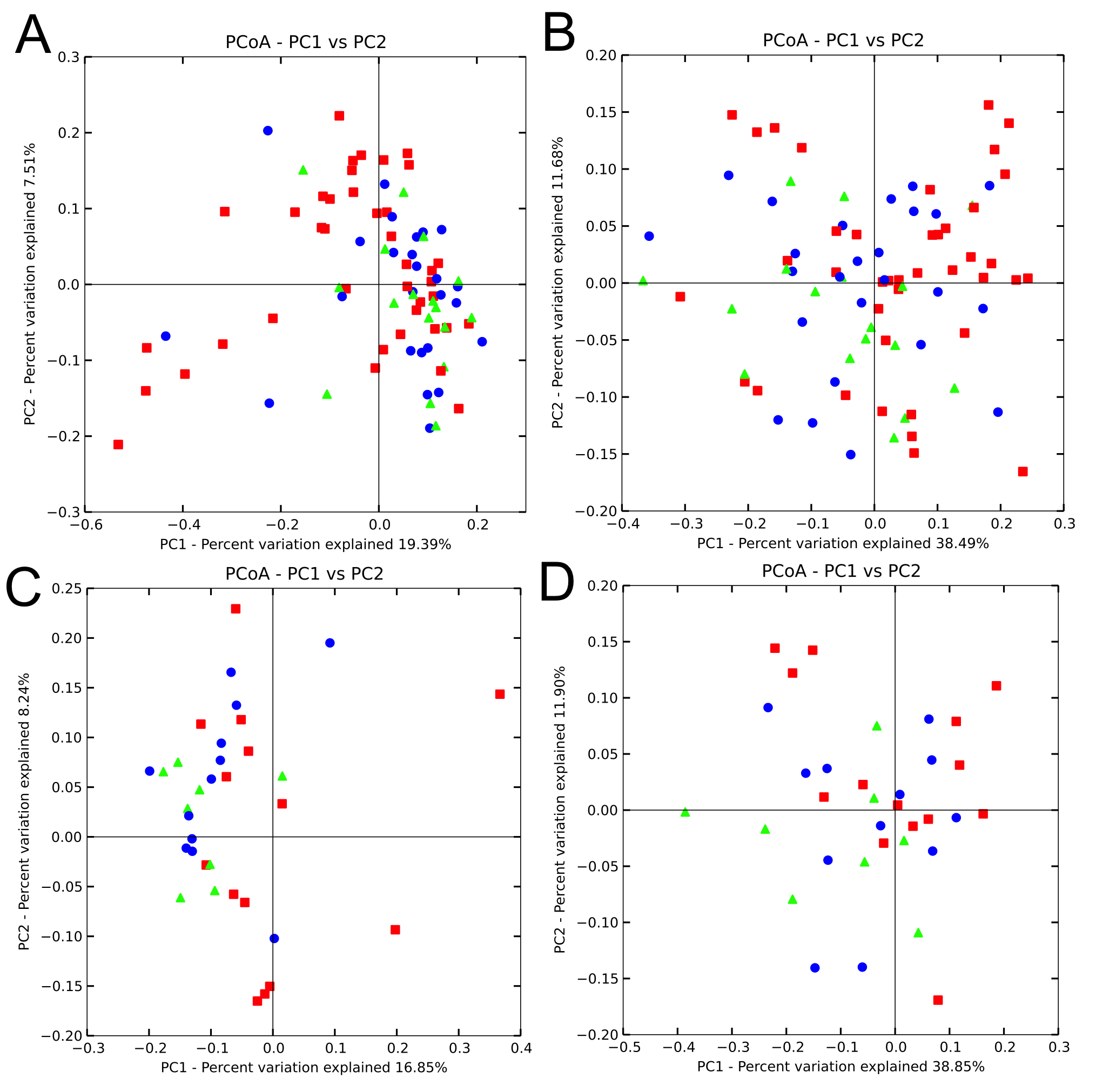

Supplement: Figure S3 — Salivary microbiome UniFrac distance data by Candida load. Salivary microbiome UniFrac distance data plotted by Candida load in all individuals (A, B) and in dentate individuals only (C, D) as unweighted (A, C) and weighted (B, D) UniFrac distance PCoA plots. Samples are colored by Candida load: green - low, blue - medium and red - high Candida load. (TIF) [file pone.0042770.s003.tif]

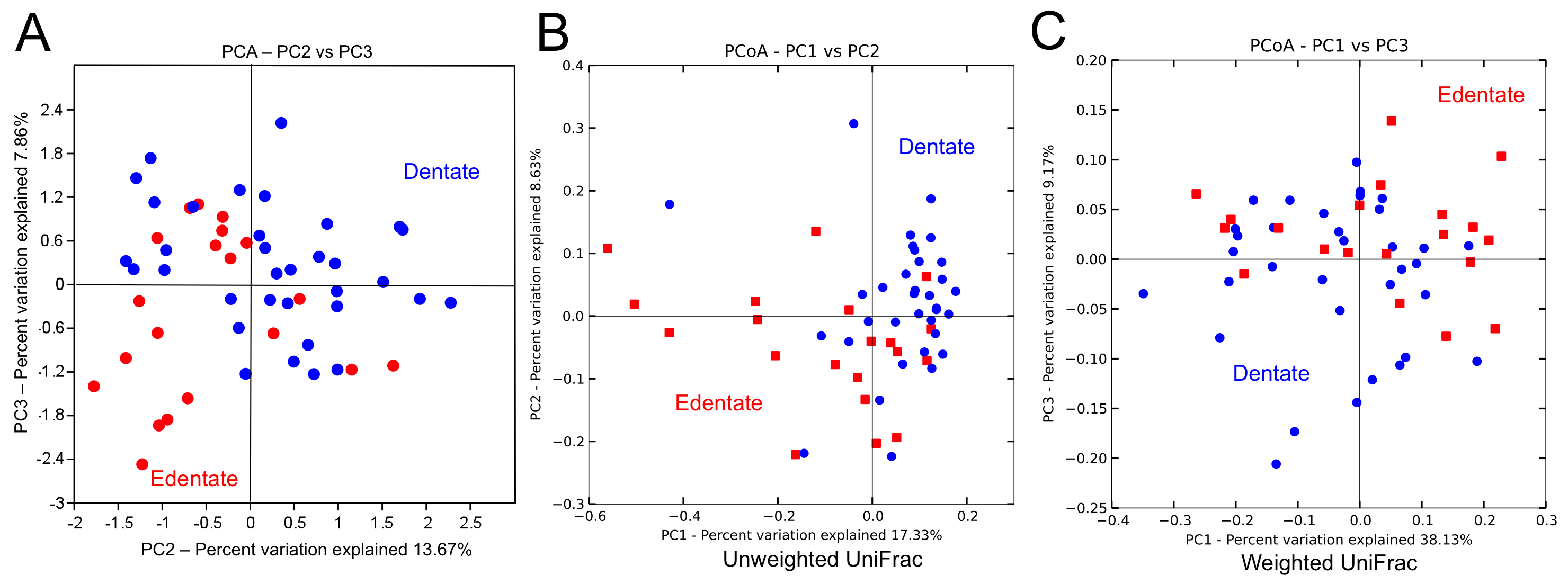

Supplement: Figure S4 — Salivary microbiome data plotted by dentition. Salivary microbiome data plotted by dentition of the subjects: A) PCA of OTU-based distances, B) PCoA of unweighted (qualitative) UniFrac distances and C) PCoA of weighted (quantitative) UniFrac distances. Samples are color-coded by the dentition status of the subjects: dentate (N = 34) – blue, edentate (N = 20) – red. (TIF) [file pone.0042770.s004.tif]
