# Supplementary material for: The Relation between Oral Candida Load and Bacterial Microbiome Profiles in Dutch Older Adults
Source: PLoS One. 2012 Aug 10;7(8):e42770. doi: 10.1371/journal.pone.0042770 (PMC3416775; doi:10.1371/journal.pone.0042770)
Supplement: Table S1 — Relative abundance of reads and number of OTUs per phylum. (DOCX) [file pone.0042770.s005.docx]

**Supplementary table S1.** Relative abundance of reads and the number of OTUs per phylum.

| Phylum | Relative abundance (%) | Nr of OTUs |
| --- | --- | --- |
| Firmicutes | 56.8 | 100 |
| Actinobacteria | 20.3 | 47 |
| Bacteroidetes | 13.7 | 66 |
| Proteobacteria | 7.4 | 37 |
| Fusobacteria | 1.2 | 14 |
| TM7 | 0.38 | 7 |
| BD1-5 | 0.08 | 1 |
| Spirochaetes | 0.04 | 7 |
| Unclassified bacteria | 0.04 | 13 |
| Tenericutes | 0.02 | 4 |
| SR1 | 0.02 | 2 |
| Synergistetes | 0.002 | 1 |
| OD1 | 0.002 | 1 |
