# Supplementary material for: The Relation between Oral Candida Load and Bacterial Microbiome Profiles in Dutch Older Adults
Source: PLoS One. 2012 Aug 10;7(8):e42770. doi: 10.1371/journal.pone.0042770 (PMC3416775; doi:10.1371/journal.pone.0042770)
Supplement: Table S2 — Relative and absolute abundance of Candida (ITS gene) in saliva samples. (DOCX) [file pone.0042770.s006.docx]

| **Table S2.** Relative and absolute abundance of Candida (ITS gene) in saliva samples. | | | | |
| --- | --- | --- | --- | --- |
| **#SampleID** | **% Candida (ITS/16S)** | **Candida CFU/ml** | **Candida load** | **Dentures** |
| LASA_100 | 0.05 | 1.37E+04 | Medium | Own_teeth |
| LASA_101 | 2.52 | 9.47E+05 | High | Full_dentures |
| LASA_102 | 0.02 | 9.39E+03 | Medium | Own_teeth |
| LASA_110 | 0.003 | 3.55E+03 | Low | NR* |
| LASA_124 | 0.15 | 3.46E+04 | High | Partial_dentures |
| LASA_133 | 0.05 | 4.44E+04 | Medium | Partial_dentures |
| LASA_137 | 0.43 | 3.92E+04 | High | Partial_dentures |
| LASA_145 | 0.05 | 7.10E+03 | Medium | Partial_dentures |
| LASA_148 | 0 | 0.00E+00 | Low | Own_teeth |
| LASA_149 | 0.28 | 6.94E+04 | High | Partial_dentures |
| LASA_150 | 0 | 0.00E+00 | Low | Full_dentures |
| LASA_157 | 0.004 | 8.76E+03 | Low | Own_teeth |
| LASA_160 | 14.5 | 3.46E+04 | High | Partial_dentures |
| LASA_161 | 0.58 | 2.16E+06 | High | Full_dentures |
| LASA_162 | 1.02 | 3.48E+05 | High | Full_dentures |
| LASA_169 | 0.18 | 1.05E+05 | High | Full_dentures |
| LASA_173 | 0.03 | 1.97E+04 | Medium | Own_teeth |
| LASA_174 | 0.14 | 6.28E+04 | High | Own_teeth |
| LASA_181 | 1440.9 | 4.33E+08 | High | Full_dentures |
| LASA_186 | 0.08 | 3.46E+04 | Medium | Partial_dentures |
| LASA_197 | 0.03 | 4.59E+03 | Medium | Own_teeth |
| LASA_198 | 0.12 | 2.60E+04 | High | Partial_dentures |
| LASA_199 | 0.004 | 7.10E+03 | Low | Full_dentures |
| LASA_200 | 0.02 | 7.10E+03 | Medium | Own_teeth |
| LASA_204 | 0.21 | 6.87E+04 | High | Partial_dentures |
| LASA_205 | 0.05 | 3.85E+04 | Medium | Own_teeth |
| LASA_208 | 0.12 | 6.93E+04 | High | Full_dentures |
| LASA_209 | 4.19 | 4.71E+05 | High | Full_dentures |
| LASA_210 | 0.46 | 4.38E+05 | High | Own_teeth |
| LASA_211 | 0.02 | 7.10E+03 | Medium | Own_teeth |
| LASA_214 | 1.78 | 3.48E+05 | High | Own_teeth |
| LASA_216 | 1.88 | 9.02E+04 | High | Full_dentures |
| LASA_217 | 0.27 | 3.51E+05 | High | Full_dentures |
| LASA_219 | 0.49 | 3.32E+05 | High | Own_teeth |
| LASA_221 | 1.11 | 1.20E+05 | High | Own_teeth |
| LASA_222 | 0.001 | 2.46E+03 | Low | Own_teeth |
| LASA_223 | 0.002 | 7.63E+02 | Low | Partial_dentures |
| LASA_225 | 0.23 | 9.60E+03 | High | NR |
| LASA_227 | 0.40 | 4.25E+04 | High | Own_teeth |
| LASA_229 | 0.37 | 2.54E+05 | High | Full_dentures |
| LASA_231 | 0.31 | 2.97E+05 | High | NR |
| LASA_232 | 0.04 | 1.51E+04 | Medium | Partial_dentures |
| LASA_234 | 0.001 | 1.51E+03 | Low | Own_teeth |
| LASA_235 | 0.05 | 5.90E+03 | Medium | Full_dentures |
| LASA_236 | 0.003 | 4.71E+03 | Low | Own_teeth |
| LASA_241 | 0.004 | 5.69E+03 | Low | Own_teeth |
| LASA_242 | 0.27 | 4.45E+04 | High | Full_dentures |
| LASA_244 | 0.03 | 5.74E+03 | Medium | Partial_dentures |
| LASA_246 | 0.001 | 8.40E+02 | Low | Own_teeth |
| LASA_247 | 0.002 | 1.21E+03 | Low | Full_dentures |
| LASA_248 | 0.52 | 2.39E+05 | High | NR |
| LASA_251 | 0.004 | 4.80E+03 | Low | Full_dentures |
| LASA_252 | 0.01 | 7.56E+03 | Low | Partial_dentures |
| LASA_253 | 0.67 | 2.15E+05 | High | Own_teeth |
| LASA_254 | 0.01 | 9.02E+03 | Medium | Partial_dentures |
| LASA_256 | 0.20 | 2.14E+05 | High | Partial_dentures |
| LASA_257 | 0.01 | 3.79E+03 | Low | Own_teeth |
| LASA_258 | 0.003 | 4.23E+03 | Low | Full_dentures |
| LASA_259 | 1.04 | 4.03E+05 | High | Own_teeth |
| LASA_26 | 0.12 | 2.97E+04 | High | Own_teeth |
| LASA_265 | 0.73 | 1.10E+05 | High | Own_teeth |
| LASA_266 | 0.01 | 7.03E+03 | Medium | Partial_dentures |
| LASA_267 | 0.04 | 1.33E+04 | Medium | Own_teeth |
| LASA_269 | 0.02 | 7.52E+03 | Medium | Full_dentures |
| LASA_270 | 2.41 | 2.55E+06 | High | Own_teeth |
| LASA_271 | 0.10 | 2.97E+04 | High | NR |
| LASA_275 | 0.01 | 1.82E+04 | Medium | Own_teeth |
| LASA_280 | 0.04 | 2.20E+04 | Medium | NR |
| LASA_282 | 0.05 | 1.16E+04 | Medium | NR |
| LASA_287 | 0.02 | 5.19E+03 | Medium | Partial_dentures |
| LASA_315 | 0.14 | 1.15E+05 | High | Full_dentures |
| LASA_338 | 0.02 | 1.26E+04 | Medium | Own_teeth |
| LASA_339 | 0.78 | 8.72E+04 | High | Own_teeth |
| LASA_350 | 0.03 | 9.45E+03 | Medium | Full_dentures |
| LASA_36 | 0.20 | 3.96E+04 | High | NR |
| LASA_40 | 14.1 | 3.42E+06 | High | Partial_dentures |
| LASA_46 | 0.14 | 2.71E+04 | High | Own_teeth |
| LASA_50 | 0.62 | 4.43E+04 | High | Partial_dentures |
| LASA_64 | 0.003 | 3.55E+03 | Low | Partial_dentures |
| LASA_70 | 0.04 | 1.15E+05 | Medium | Own_teeth |
| LASA_94 | 0.12 | 6.92E+04 | High | Own_teeth |
| LASA_97 | 0.04 | 3.81E+04 | Medium | Own_teeth |
|  |  |  |  |  |
|  |  |  |  |  |
| * - NR - not reported by the subject in the questionnaire | | | |  |
